# Custom-Made Zirconium Dioxide Implants for Craniofacial Bone Reconstruction

**DOI:** 10.3390/ma14040840

**Published:** 2021-02-10

**Authors:** Marcin Kozakiewicz, Tomasz Gmyrek, Radosław Zajdel, Bartłomiej Konieczny

**Affiliations:** 1 Department of Maxillofacial Surgery, Medical University of Lodz, 113 Żeromskiego Str, 90-549 Lodz, Poland; tomasz.gmyrek@umed.lodz.pl; 2 Department of Informatics and Statistics, Medical University in Lodz, Pl. Hallera 1, 90-647 Łódź, Poland; radoslaw.zajdel@umed.lodz.pl; 3 University Laboratory of Materials Research, Medical University of Lodz, 251 Pomorska, 92-213 Lodz, Poland; bartlomiej.konieczny@umed.lodz.pl

**Keywords:** zirconium dioxide, custom implants, ultrahigh molecular weight polyethylene, titanium alloy, craniofacial, maxillofacial surgery, cytotoxicity, genotoxicity, bone defect treatment

## Abstract

Reconstruction of the facial skeleton is challenging for surgeons because of difficulties in proper shape restoration and maintenance of the proper long-term effect. ZrO_2_ implant application can be a solution with many advantages (e.g., osseointegration, stability, and radio-opaqueness) and lacks the disadvantages of other biomaterials (e.g., metalosis, radiotransparency, and no osseointegration) or autologous bone (e.g., morbidity, resorption, and low accuracy). We aimed to evaluate the possibility of using ZrO_2_ implants as a new application of this material for craniofacial bone defect reconstruction. First, osteoblast (skeleton-related cell) cytotoxicity and genotoxicity were determined in vitro by comparing ZrO_2_ implants and alumina particle air-abraded ZrO_2_ implants to the following: 1. a titanium alloy (standard material); 2. ultrahigh-molecular-weight polyethylene (a modern material used in orbital surgery); 3. a negative control (minimally cytotoxic or genotoxic agent action); 4. a positive control (maximally cytotoxic or genotoxic agent action). Next, 14 custom in vivo clinical ZrO_2_ implants were manufactured for post-traumatologic periorbital region reconstruction. The soft tissue position improvement in photogrammetry was recorded, and clinical follow-up was conducted at least 6 years postoperatively. All the investigated materials revealed no cytotoxicity. Alumina particle air-abraded ZrO2 implants showed genotoxicity compared to those without subjection to air abrasion ZrO_2_, which were not genotoxic. The 6-month and 6- to 8-year clinical results were aesthetic and stable. Skeleton reconstructions using osseointegrated, radio-opaque, personalized implants comprising ZrO_2_ material are the next option for craniofacial surgery.

## 1. Introduction

The most common consequence (40%) of maxillofacial structure injuries is orbital region trauma [1]. If there is no complete reconstruction at the time of primary surgical repair, this may influence the outcomes. Facial skeleton fractures are often associated with the risk of functional disturbances (double vision and ptosis) as well as aesthetic disturbances (face asymmetry and enophthalmos) [1,2]. To date, no single ideal material is available to repair these post-traumatic defects and restore function. However, three options exist as ways to shape the reconstructive material: 1. manually/arbitrarily; 2. individually on the model but only for plastic materials possible; 3. design/manufacture of individual implants using computer-assisted techniques—i.e., a final implant without model or manual shaping. This third technique is the subject of this study.

Recently, custom-made implants have been prepared by computer-assisted design (CAD) and manufacturing (CAM) for facial skeleton region reconstructions [3,4,5,6,7]. The goal of surgical reconstruction is to restore function and facial appearance. It is obtained by removing herniated tissues from the fracture gap, supporting the soft tissues and reconstructing the bone to prevent the return of the hernia. The most important issue in orbital reconstruction is obtaining the correct shape of the bone surface supporting the eye globe and skin, because an inaccurately corrected orbital volume causes unpleasant aesthetic consequences (enophthalmos). However, the type of material used, its properties, the correctly designed shape of the implant, the appropriate surgical technique and the condition of the patient are all crucial for success. The material applied should be biocompatible and allow fixation to host bone by screws, wire, suture or adhesive. It should not potentiate the growth of microorganisms or promote resorption of the underlying bone or deformation of adjacent tissues. Furthermore, radiopaque materials should be used to allow radiographic evaluation [8]. Visibility in radiological examination allows confirmation of correct implantation. In the case of implant malposition detected in the intraoperative imaging scan, it allows for immediate correction of the position, avoiding a second surgery. The search continues for the ideal implant and reconstructive material, and particularly osseointegrated materials, such as titanium. Titanium is currently the standard material in craniofacial reconstructions; another material is zirconium dioxide.

Zirconium dioxide (zirconia) is a ceramic material with physical properties that have long been appreciated in the space industry to cover shuttles and manufacture medical devices. In 1969, zirconium dioxide (ZrO_2_) material was first studied by orthopedics for hip head replacement [9]. Since 1985, ZrO_2_ has been applied to make the artificial head of the hip [10]. Its unusual break resistance (2000 N), compression resistance (2000 MPa) and biocompatibility have found their application in implantology to reconstruct missing teeth as well as hips, knees, shoulders and wrists [9]. Its mechanical properties are very similar to those of metals. MgO, CaO or Y_2_O_3_ interstitials are being added for greater molecular stability. The most studied combination is yttrium-stabilized zirconia, also known as tetragonal zirconia polycrystals. ZrO_2_ stabilized with Y_2_O_3_ has better mechanical properties than other combinations [9]. Despite a much more difficult process of sintering, this form of zirconia is considered for medical applications.

Clinically, ZrO_2_ has several advantages over titanium. The inflammatory reaction to ZrO_2_ is much lower than that to other restorative materials, even titanium [10,11]. Evaluating soft tissues in contact with titanium and ZrO_2_ reveals a higher inflammatory reaction around titanium [12]. Additionally, in the case of titanium implanted during the treatment of trauma-related effects, changes in antioxidant defense and increased synthesis of the products of oxidative damage of proteins and lipids indicate the body’s adverse response to the introduction of titanium material [13]. Furthermore, the level of bacterial products measured using nitric oxide syntheses is higher on titanium than on zirconium dioxide [9]. The use of zirconium dioxide in craniofacial reconstruction has not been described in the literature thus far and appears very promising.

The authors aimed to evaluate the cytotoxicity and genotoxicity of zirconium dioxide material as well as present a new application of that material for craniofacial bone defect reconstruction.

## 2. Materials and Methods

In vitro-tested materials were formed in 2 mm-thick disks with diameters of 10 mm. Titanium alloy grade 23 powder (ISO 5832-3:1996 for selective laser melting) [14] was used in an SLM 250 machine (SLM Solutions GmbH, Lübeck, Germany). The thickness of the added layers in the implant was 30 μm (Z-axis move), and the construction speed was 25 cm^3^/h. Next, the disks were subjected to annealing at 700 °C for 1 h in an argon atmosphere, slowly cooled down in a furnace and subjected to the tempering process. Ten disks produced in this way created a comparison group as a standard material for craniomaxillofacial surgery.

The next comparison group consisted of 10 disks milled from ultra-high-molecular-weight polyethylene (UHMW PE; [15,16,17]) in a computer numerical control (CNC) process [3] by Speed Hawk 650 (OPS-Ingersoll Funkenerosion GmbH, Burbach, Germany).

Zirconium dioxide disks were milled from prefabricated blocks (the substrate block Prettau Zyrconia of dimensions 125 mm × 70 mm × 20 mm was delivered by Zyrkonzahn Worldwide, Gais, Italy, https://www.zirkonzahn.com/ (accessed on 10 January 2021)). Twenty percent enlarged substrate disks were CAD designed, and CNCs were milled using a 5-axis milling machine [3] by Speed Hawk 650 (OPS-Ingersoll Funkenerosion GmbH, Burbach, Germany). Subsequently, sintering of substrate disks was performed at 1450–1500 °C according to the manufacture of zirconium dioxide substrate. The observed shrinkage was 20% in all directions (the process is described in Section 2.3). Next, the alumina particle air-abrasion series (ZrO_2_+ air-abrasion: 10 disks) and no surface-modified series (ZrO_2_: 10 disks) were collected. Zirconium dioxide disks were air-abraded using 50 µm Al_2_O_3_ particles (Cobra, Renfert GmbH, Hilzingen, Germany) with a pressure of 3 bar from a distance of 10 cm for 10 s.

Experimental disks were placed, in batches of 10, into a solution of a washing and disinfecting preparation (Aniosyme DD1, Laboratories Anios, Pavé du Moulin, France). The solution was obtained by taking 5 mL of the preparation and dissolving it in 1 L of distilled water. The disks were completely submerged below the liquid level. The ultrasonic cleaner container (Ultrasonic Cleaner CD-4820, Zhengzhou Smile Industrial Co Ltd., Zhengzhou, China) was covered with a lid, and the cleaner was started (washing time: 10 min. disinfection time: 20 min). The disks were then removed from the solution, rinsed with distilled water and dried. The packing and autoclavation process (at a temperature of 134 °C and 2 atm. pressure in a 1-h cycle) preceded in vitro tests [18].

Later, an in vivo experiment was performed only in ZrO_2_ implants. After sintering, the implants were not subjected to airborne particle abrasion.

### 2.1. Cytotoxicity Study

The disks of the materials tested were placed on a 12-well cell culture plate, and then 1 × 10^5^ Saos-2 cells/mL/pit in 2 mL of growth medium were seeded on them. The negative control was constituted by cells cultured without the tested materials on an unmodified surface of the plate. The positive control was constituted by cells cultured with medium containing 1% Triton X-100 (as a maximal toxic environment inducing cell membrane lysis). After 48 h of incubation, the medium was collected from the culture, conditioned and centrifuged at 600× *g* for 5 min, and the supernatant was used to determine lactate dehydrogenase (LDH) activity with the protocol proposed by the manufacturer (Sigma: No. TOX7; Merck KGaA, Darmstadt, Germany). LDH activity in the supernatant measured the cytotoxicity of the tested material to Saos-2 cells.

### 2.2. Genotoxicity Study

To test the genotoxicity, the test materials were placed under sterile conditions in 12-well plates, and then the Saos-2 cells were seeded at a density of 5 × 10^4^/well in 2 mL of growth medium. Saos-2 cells cultured without the tested materials were the negative control—i.e., control (−). The positive control was performed by cells incubated for 24 h with mitomycin C at a final concentration of 0.8 µg/mL (as a standard genotoxicity agent)—i.e., control (+). After 24 h of cell growth, cytochalasin B at a final concentration of 3 µg/mL was administered to all samples to inhibit cytokinesis. After 48 h of cellular DNA incubation, Hoechst 33342 (catalog number: H3570; Cell Culture (Core Cell Culture): Thermo Fisher Scientific Polska; Warsaw, Poland) was added (0.5 µg/mL) for labeling and imaging using an InCell Analyzer GE Healthcare automated microscope (Global Life Sciences Solutions Poland Sp. z.o.o., Warsaw, Poland). The resulting images were analyzed automatically using InCell Analyzer GE Healthcare software (version 7.0, Global Life Sciences Solutions Poland Sp. z.o.o., Warsaw, Poland).

Next, scanning electron microscopy of the samples was performed: the output samples were visualized without prior preparation, and the sample after cell culture was fixed (2.5% glutaraldehyde and 2% paraformaldehyde in 0.1 M phosphate buffer at pH 7.4 for 24 h), dehydrated and finally dried at room temperature. The samples were visualized using a Phenom ProX scanning electron microscope (Life Technologies Polska Sp. z o. o., Warsaw, Poland) in a back-scattering electron detector. An accelerating electron voltage of 5 kV and magnifications of 250×, 500×, 1000×, 2000× were used. In material samples after culture without cells, a 10-kV voltage and 5000× and 10,000× magnifications were also used.

### 2.3. Clinical Application of the Zirconium Dioxide Implant

The material used to manufacture personalized implants was the same as that used in the in vitro experiment. Fourteen consecutive and delayed cases were included in this study after approval by the university ethical board (RNN/141/12/KB). Only post-traumatic cases were referred several months after the injury (old fractures and delayed treatment). All of them had previously undergone surgical treatment, including bleeding halt, wound dressing, neurosurgical or ophthalmological management, and in half of the orbital wall fractures, partial bone defect reconstructions were performed.

The patient’s face was three-dimensionally scanned using a 3dMDface System (3dMD, Atlanta, GA, USA) immediately before surgical treatment and 6 months postoperatively when the soft tissue configuration was stabilized. The duration of face-scanning was 100 ms. As the pre- and postoperative data were collected, they were superimposed on each other, and a horizontal plane of tragus and tip of the nose was appointed (to capture changes in the skin surface position in the posterior-anterior direction). The plane was shifted to cut the reconstructed region (zygomatic, orbital, forehead), and lines marking the site of cutting skin surface position pre- and postoperative by the horizontal plane were recorded. A series of measures (5–7 counts) between the pre- and postoperative soft tissue positions were made after establishing the two lines in place of skin surface sections of superimposed 3D face scans. Next, the average posterior-anterior soft tissue shift in millimeters was calculated.

High-resolution, helical computer tomography (CT) was performed in all patients (64-raw detector; 512 × 512 matrix; 0.6 mm scans). Next, facial skeleton segmentation, mirrored images of intact sites, superimposition intact on affected sites, and analysis of symmetry were performed (Geomagic Studio, Geomagic Corp., Morrisville, NY, USA). Finally, surfaces together with 3D coordinates were exported to CAD software (Solid Works, Dassault Systèmes SolidWorks Corp., Waltham, MA, USA) to design 20% enlarged substrate implants and computer numerical controls milled by a 5-axis milling machine [3] from zirconium dioxide. Before that, the maxillofacial surgeon had the opportunity to indicate the extension and dimensions of the implant and inspect and, if needed, reshape the virtual implant. It was extremely important to focus closely on the extension of the final reconstruction to avoid making the implant too small or too rough. Next, the virtual implant was ready to be approved by the maxillofacial surgeon. The substrate material cost was approx. EUR 150, and a set of multiple-use burs was approx. EUR 200. It was mandatory to design and mill fixing screw holes in the implant. The main part of the implant was milled by a 4 mm cutter. The milling began from the center and finally reached the edges of the implant (to avoid cracks). The cutter rotation speed was 6000 rpm, with a movement of 16.7 mm/s. Safety fracture lines (grooves) were milled by a 2 mm cutter with a rotation speed of 10,000 rpm in orbital wall implants. The grooves reached a depth of 300 μm. The maximum thickness of the pre-sintered orbital implant was 0.85 mm, and the minimum thickness was 0.55 mm. In zygomatic implants, thickness depended on the need for zygomatic region augmentation (maximal 5–7 mm). When the implant was reconstructed, the calvaria was 2 mm thick in this area. Next, perforations for the fixing screws were performed. The final perimeter cut, which released the implant from the substrate block, was performed by a 2 mm cutter with a high speed of 12,000 rpm. Next, sintering of the substrate implant was performed at 1450–1500 °C. Further correction might have been performed intraoperatively using diamond burs and sterile water. The observed shrinkage was 20% in all directions.

Because of the results of the genotoxicity tests, only zirconia not subjected to air abrasion was used, and no surface modifications were made. Next, implants were laser-beam-marked, uncontaminated, sterilized and delivered to the operating theatre.

The effect of the injury was quantified using the orbital destruction intensity scale (ODI) in the included patients [18]. This scale simultaneously describes reconstructive needs. The scale is described as follows: 1. site of destruction—floor, i.e., one wall (1 W); 2. floor + one additional wall medial or lateral, i.e., two walls (2 W); 3. floor + one orbital margin, i.e., one wall and one orbital margin (1 W + 1 M); 4. floor + one additional wall + one margin (2 W + 1 M); 5. floor + one additional wall + two margins (2 W + 2 M); 6. floor + two additional walls + one margin (3 W + 1 M); 7. floor + one or two additional walls + two margins (3 W + 2 M); 8. floor + two or three additional walls + more than two orbital margins (3–4 W + 3–4 M).

All the patients were subjected to surgery using general anesthesia. A transconjunctive [19] approach was performed in orbital wall cases [5,6], a transoral by upper fornix approach in zygomatic bone deformations and a skin approach either through the eyebrow scar or coronal [1] as a bone defect or deformity extending to the calvaria. After exposure to the bone defect or deformed bone surface, the implant was inserted and stabilized in position. The onlay implants were positioned to reconstruct the injured orbital wall and screwed to the lower orbital rim by titanium screws (1.5 mm diameter and 4.0 mm length). The same screws were used for the cranioplasty case. The custom zygomatic and upper orbital rim implants were positioned in proper places and fixed onto the surface of the bone using titanium screws (1.5 mm diameter and 12.0 mm length). The final implant position was easy to find because of patient-specific surfaces in the implant contacting the affected bone shape. Only one position existed where the implant touched all surrounding bone surfaces. The patients were then followed up in an outpatient department for a minimum of 6 years. After 6 months, a repeat photogrammetric study (3dMD) was performed.

Statistical evaluation was performed using Statgraphics Centurion Version 18.1.12 (StarPoint Technologies Inc., Addison, TX, USA), which included summary statistics, analysis of variance (to check the material cytotoxicity and genotoxicity, influence of the implant on the region of reconstruction or influence of sex on facial soft tissue shift) and linear regression analysis (to evaluate the relationship of age or ODI scale to facial soft tissue shift). Statistical significance was determined as *p* < 0.05.

## 3. Results

### 3.1. Cytotoxicity Results

Cytotoxicity studies using lactate dehydrogenase enzyme proved no statistically significant difference in cell survival on the surface of the tested materials (Table 1).

### 3.2. Genotoxicity Results

In the micronucleus test measuring genotoxicity, micronuclei are defined as chromatinous structures inside the cytoplasm. These structures are formed because of damage to the split spindle, leading to the separation of one or more smaller nuclei. Micronuclei can be formed from the whole chromosome or because of the separation of an acentric fragment of the chromosome. The results are presented below in Table 2.

**Figure 1:**
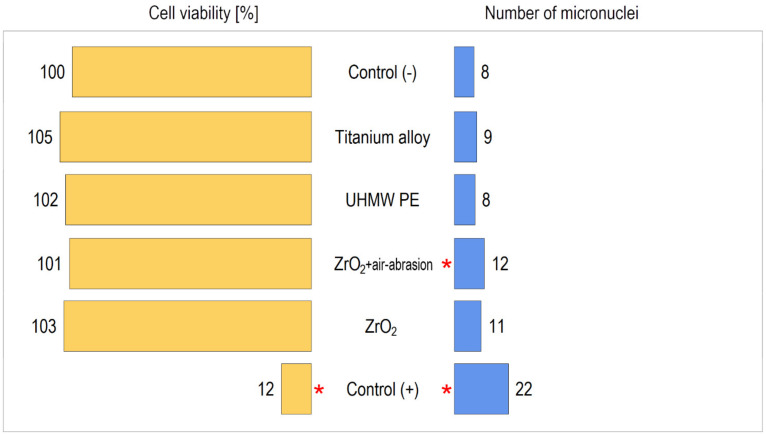
Cell viability (higher values are better—i.e., less cytotoxicity) and number of micronuclei (lower values are better—i.e., less genotoxicity). Control (+) is extremely cyto- and genotoxic (the asterisk indicates a significant difference from Control (−)), while Control (−) is the most neutral. The tested materials are not cytotoxic. Alumina particle air-abraded ZrO_2_ implants (the asterisk indicates a significant difference from Control(−)) is significantly more genotoxic than the other test materials, including intact ZrO_2_.

The alumina particle air-abraded ZrO_2_ samples showed genotoxicity characteristics (*p* ˂ 0.01; one-way analysis of variance (ANOVA)). In the microscopic image, the cells growing on the examined surfaces changed morphology. The number of micronuclei per 100 binuclear cells was the largest of the materials used in the study (Figure 1).

### 3.3. Clinical Treatment with a Zirconium Implant in Patients

Fracture of a pre-sintered implant was present in one case during milling caused by a bur that was not sufficiently sharp. The next copy of the implant was manufactured using a new bur set and a substrate block based on the same milling program.

All the surgical operations (Table 3) were performed using custom zirconium dioxide implants. During one implantation, the margin of the implant border cracked in the orbital rim area (case #12) and was not palpable through the skin because the lost part was much thinner than 0.5 mm. The medial rectus muscle wrapped the implant border during medial orbital wall reconstruction, affecting eye globe motility in one case (case #2). A second surgery was required to reposition the muscle on the following day; the position for the implant was not changed. In the other 13 of 14 patients, the postoperative course was without complications. The duration of one drainage case was two days (case #13). Problems in zygomatic implant positioning were noted twice (intraoperatively), likely caused by insufficient bone exposure in the area of the zygomatic arch and frontal process of the zygomatic bone; these issues were resolved after more spacious subperiosteal preparation. No drainage was used except in the coronal approach case. The hospital stay was 3–7 days, and the wounds were mostly healed. The stitches were removed from the eyelid on the fifth day and from the mucosa on the seventh day. The skin was excluded from the coronal approach, in which the stitches were removed on the tenth day. After the transconjunctival approach, absorbable 7/0 sutures were left for resorption. The postoperative outcomes returned facial symmetry. The patients regained a normal appearance and showed improved vision function. The control CTs were acquired one month postoperatively using the same protocol as preoperative CT and showed the location of the implants because zirconia is visible under X-ray examination (radio-opaque). The implant position was determined according to the plan and was stable in all cases for at least 6 years of follow-up (annual follow-up examinations). No long-term clinical complications were observed.

Examples of each reconstructive application (orbital wall, zygomatic deformation and upper orbital rim with forehead) and preparations of zirconium dioxide individual implants are presented in Figure 2, Figure 3, Figure 4, Figure 5, Figure 6, Figure 7, Figure 8 and Figure 9.

The augmentation by the custom ZrO_2_ implant was stable at the follow-up in all cases. The average soft tissue shift in the augmented region was 3.79 ± 2.02 mm (range: 1.27–7.84 mm). The soft tissue improvement was at a level slightly below statistical significance (*p* = 0.0564) related to patient age (correlation coefficient = −0.52; R^2^ = 27%) but was significantly more visible (*p* < 0.05, F = 5.92) in the cases of the reconstruction of zygomatic deformation (4.87 ± 1.82 mm) and forehead (5.87 ± 2.79 mm) than in orbital wall defect treatment (2.42 ± 0.87 mm). The same soft tissue shift was observed in both sexes (*p* = 0.9032). The correlation coefficient equaled 0.68, indicating a moderately strong relationship between the soft tissue shift and ODI scale (*p* < 0.01; R^2^ = 49%); in more extended damage, a higher improvement in soft tissue was noted (Figure 10).

## 4. Discussion

Titanium alloys are currently the standard in craniomaxillofacial surgery. Alloys grade 5 or 23 are mainly used, but they are not ideal implantation materials for humans [20]. Titanium alloy is implanted into the bone; after some time, it passes into the surrounding soft tissues and reaches high concentrations there. The number of micronuclei in the cells of patients with titanium implants is higher than that in patients without titanium implants. However, this assessment of cytogenetic activity [21] is not statistically significant (as in this study). An elevated titanium ion content in tissues inhibits cell proliferation by inhibiting enzymatic activity [22], oxidative stress [13] and nitrosative stress [23]. At the same time, zirconium oxide ceramics cause less inflammation after implantation than titanium alloy [24]. Surprisingly, in this study, test implants made of alumina particle air-abraded ZrO_2_ showed toxicity. Perhaps the air-abrasion of the ZrO_2_ implant by aluminum oxide leaves abrasive nanoparticles embedded in the surface [25], and these grains increase genotoxicity, highlighting the disadvantage of using ceramics [26] and making the biological effect similar to the adverse effects of using metal alloys. Higher concentrations of Al_2_O_3_ affect the cells they contact [27]. On the one hand, the search for new materials resulted in the introduction of implants sintered from metal powders (such as the titanium alloy group in this experiment) 10 years ago. Thus, such plates entered maxillofacial surgery; however, the technology of their manufacture makes them have a rough surface. Such a surface is exposed to bacterial invasion by creating a biofilm [28]. On the other hand, fused ceramic surfaces can be smoother. Hence, interest in ceramics for zirconium oxides has increased. After the in vitro tests, these ceramic implants should not be subjected to air abrasion by Al_2_O_3_. Thus, personalized ZrO_2_ implants with an unmodified surface are safe. Perhaps in the future, other useful methods of surface modification than simple alumina particle air-abrasion will be developed. Alternatively, other types of zirconia will be used, as well as other stabilizers, other grains, and pressure pre-processing of powder.

Ceramic implants made of zirconium oxide have been used to treat people for over 50 years [29,30]. One of their disadvantages is that they break in places of the skeleton where they are mechanically loaded [31]. This disadvantage is not present in the application proposed in this study for craniofacial surgery. Neither the orbital walls nor their surrounding areas nor the calvaria carry loads comparable to knee or hip joint reconstructions.

Reconstruction of the facial skeleton is a challenge for surgeons due to difficulties with the maintenance of long-term aesthetic and functional effects. The use of different techniques and materials has changed the way surgeons proceed with patients who have post-traumatic complaints [32]. In 1967, Converse et al. [33] reported their 10-year experience with late correction of orbital fractures. They emphasized the problems with secondary correction of established diplopia and enophthalmos. In this study, all 14 patients fell into this challenging category described by Converse. Many other authors [34,35,36,37] have pointed out that restitution of hard and soft tissues might be very difficult to obtain in delayed treatment. The use of different materials arises from the mentioned problem. Autologous grafts are the most natural way of reconstructing damaged skeletal structures, but an intimate correlation has been confirmed between resorption and increasing the level of clinical enophthalmos [38]. The use of alloplastic materials has many advantages, such as stability of volume and lack of perioperative morbidity, as well as drawbacks, such as high costs and lack of sufficient endurance to static and dynamic stresses [39,40]. Finally, the shape of the reconstruction material is important. Converse had no CAD/CAM support so it could only model the material manually. This approach began to change 25 years ago. In 1998, Hoffmann et al. proposed the application of dental prosthodontic procedures to develop an individual facial skeletal implant made of metal oxide ceramic [1]. At that time, the advanced engineering methods applied today were not available [5,6,41]. Since then, computer programs and computer technology have developed new tools that are much more powerful. However, Hoffmann’s team proposed and clinically utilized the analog coping method of an implant model into ceramic material. Stereolithographic models were used to design the implant. This method was also used in Lieger et al.’s [4] surveys. After copy-milling a resin template with a commercially available dental unit, the prefabricated implants were inserted to reconstruct the lamina papyracea, zygomatic complex, orbital lower wall and infraorbital rim. The clinical results were very satisfying. This is the first attempt to use individual ceramic implants in maxillofacial surgery.

Currently, only two large medical companies, DePuy Synthes and KLS Martin, and a few smaller manufacturers, produce custom implants for doctor demand. Polyetheretherketone or titanium are the available materials (http://www.oblparis.com/porousiti?lang=en (accessed on 10 January 2021), http://www.chm.eu (accessed on 10 January 2021)). These patient-contoured implants are mainly offered for neurosurgical reconstruction, probably due to simple segmentation of the thick bones of the cranial vault (orbital wall segmentation is labor-intensive because of paper-thin bone). The price level of that service and product is significant—i.e., more than EUR 2500 per case. Unfortunately, zirconium dioxide ceramics (ZrO_2_) have only recently been developed for dental applications [42], and insufficient clinical evidence exists to support any other skull application—i.e., facial skeleton augmentation. Various ceramic implant systems made of yttria-stabilized tetragonal zirconia polycrystals have become commercially available in recent years [43]. ZrO_2_ dental implant studies presented a success rate ranging from 84% after 21 months to 98% after 1 year [44]. The material used here was partially stabilized zirconia with Y_2_O_3_ in amounts of 4.95–5.36 wt%. The grain size is relatively high (0.35–0.79 μm) [45,46], which may increase the pore size. The roughness of the material after the sintering process is R_a_ = 0.11 ± 0.01 [45]. Therefore, it seems that the material for the ceramic individual implants in this study was chosen correctly.

Presently, a full digital/virtual method of implant production is possible [5,6,7,41]. This method improves the accuracy of the procedure, simplifies and accelerates the manufacturing process and reduces costs. It is possible to use implants as thin as 0.55 mm or thoroughly modulated thick implants [3]. The proper repositioning of the eye globe is the main purpose of the surgical procedure in enophthalmia and diplopia treatment, which requires a proper orbital wall implant. With current advancements in cosmetic surgery and maxillofacial surgery, reconstruction of missing bone structures has important functional and aesthetic aspects. Thus, custom ZrO_2_ implants are a helpful invention that may improve the reconstruction of post-traumatic injuries of the cranio-maxillo-facial skeleton, leading to the restoration of lost facial symmetry.

**Figure 4:**
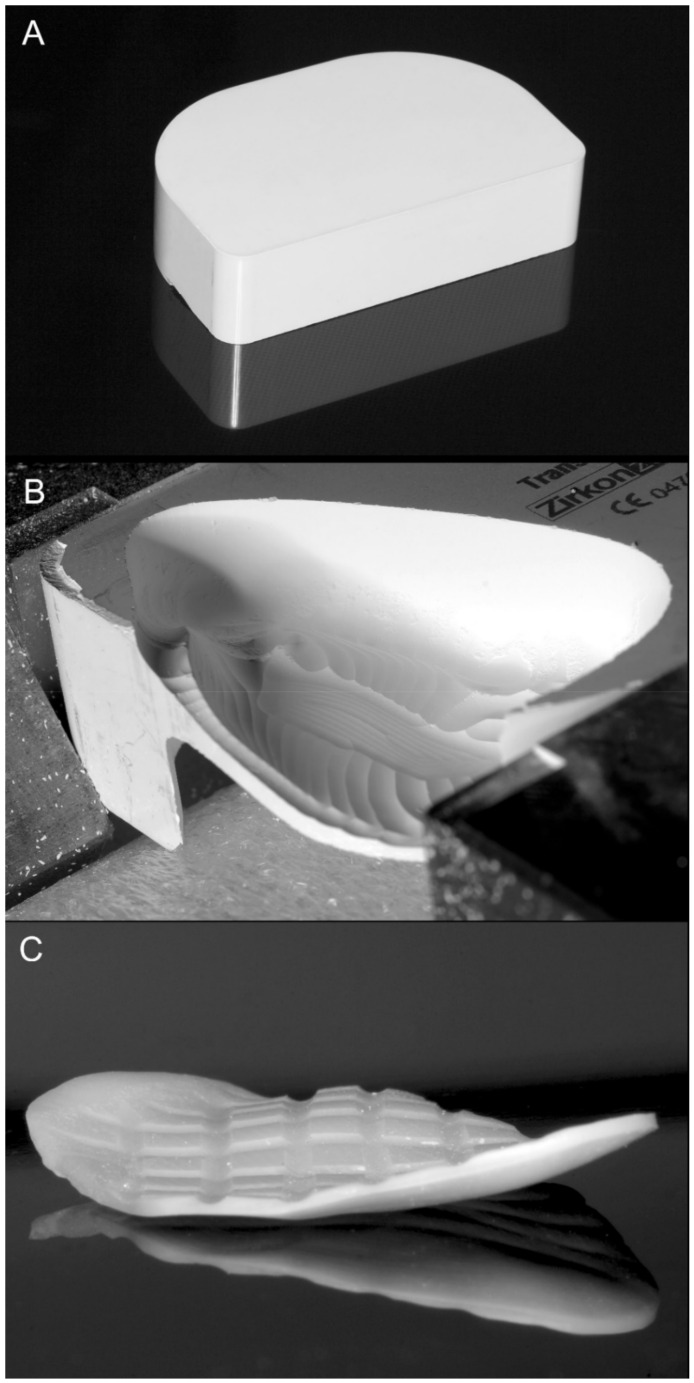
Computer-assisted manufacturing (CAM). (**A**), Substrate zirconium dioxide block. (**B**), Implant during milling. (**C**), Implant after sintering before cleaning and sterilization. The crisscross grooves in the surface of the implant facilitate control fracture of the implant in a potential future facial injury incident.

**Figure 5:**
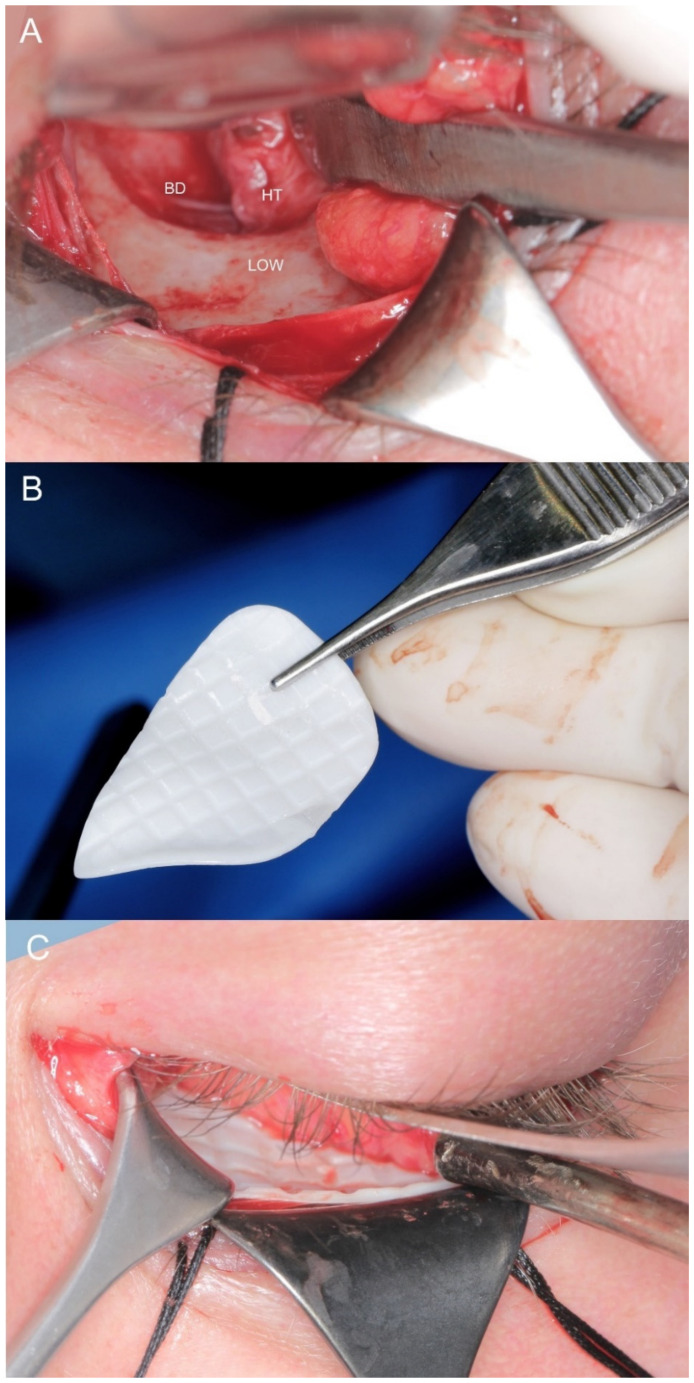
Intraoperative view. (**A**) The transconjunctival approach exposes the operating field in the left orbit (LOW—lower orbital wall; BD—bone defect; HT—herniated tissue). (**B**) Zirconium dioxide implant before insertion into the orbit. (**C**)—The patient-specific implant reconstructs the lower orbital wall.

**Figure 10:**
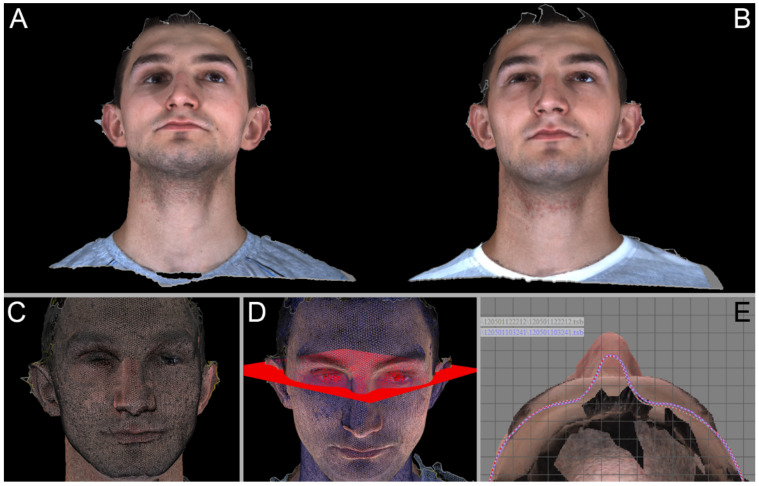
Photogrammetry. Evaluation of the treatment results. Right site, lower orbital wall reconstruction. Upper Row: Unedited, pre- (**A**) and postoperative (**B**) 3D face scans. Lower row: Superimposition of pre-post operational digital meshed constructed based on 3D face scans (**C**). Horizontal plane (**D**) utilized for calculating the improvement in face symmetry. Top view (**E**) of superimposed 3D face scans with red surface marking line of cutting skin surface position pre- and postoperatively by the horizontal plane.

Custom ZrO_2_ implant applications seem to be solutions that have many advantages and lack the disadvantages of other materials. Compared with auto- and allografts, it does not resorb and accidentally deforms during and after surgical operation [47,48]. In contrast to polymer implants (silicon, polyethylene and simple polyether ether ketone, zirconium dioxide has excellent visibility on X-ray examination and the ability to osseointegrate, a function that is most desired in bone reconstructions [44]. The additional merit of this material is the price, which is EUR 150 for one block (sufficient to make two orbital implants or one cranioplastic implant) and the possibility of repairing post-sintered implants with porcelain. This material also has some disadvantages, such as stiffness and fragility, which cause technical difficulties and the danger of uncontrolled fracture in the case of a secondary injury of the head (therefore, it was proposed as a modification structure). After sintering, its stiffness is much higher than that of cortical bone. In the cases of floor orbit reconstruction, the authors designed crisscross grooves on the surface of the implant to facilitate controlled fracture of the implant in the next potential facial injury incident (Figure 4 and Figure 5). Otherwise, the thin and rigid implant might have behaved like a blade that jeopardizes the fragile content of the orbit and cranial fossa.

The advantages of zirconium dioxide implants may become more widespread with the already available additive technique (Lithoz GmbH, Wien, Austria), which facilitates many technical issues in the manufacture of individual craniofacial implants. Ceramic 3D printing enables printing with two materials simultaneously, and the LED-driven machine (CeraFab Multi 2M30) allows the production of implants containing combinations of ceramics (zirconia, alumina, tricalcium phosphate and silicon nitride), metals or polymers. The dual approach is presented at www.lithoz.com/en/3D-printing/medical-dental, in which a cage made of high-strength ZrO_2_ provides support during the healing phase and where the inner volume of the implant comprises, for example, bioresorbable beta-tricalcium phosphate. Creating implants with adjustable/designed porosity is an interesting possibility and would be a significant advance in the development of ceramic individual implants for maxillofacial surgery. Additionally, no scientific publications are available using this technique for craniofacial reconstructions; it has only been used on scaffolds in animals [49].

Despite these benefits, after accurate skeletal reconstruction, soft tissue should be inspected. In posttraumatic cases, some asymmetry caused by scars, skin thinning, fat loss or other deformations might exist. In those cases, adjuvant treatment is necessary. Currently, the best opportunity is structural fat grafting [50,51,52].

The advantages of the proposed individual implants are as follows:

1. Anatomical symmetry
2. No donor site morbidity
3. Easy positioning—i.e., no need for long-lasting maneuvers inside the operating field
4. No resorbable implant—i.e., long-time stale shape
5. Stiff reconstructions
6. Not only surface, but volume-restoring implant
7. Osseointegration
8. Better thermal conductivity than titanium
9. Visible in roentgenological imaging
10. Not affect magnetic resonance imaging (excluding titanium screw artifacts)

Disadvantages:

1. Time needed for design and manufacture
2. Surgeon involvement in design
3. Limited subsequent implant modification during surgery
4. CT artifacts because the implant is thick on follow-up examination
5. Limited volume of the substrate material block for cranioplasty.

Practical issues should be indicated in these new types of patient-specific implants. Thin and long parts of the implant face cracks before and after sintering. One case of fracture of a thin margin (<0.5 mm) of one implant was observed. Complex shapes and screw holes may require the application of 5-axis milling machines, which are less available than 3-axis devices. Finding the final implant position during surgery is difficult because the approach that is performed is too narrow. It is also against the will of surgeons who want to perform esthetic and invisible approaches. Finally, implant drilling cannot be performed during surgery. Therefore, active participation of the surgeon during the implant design is necessary.

Second, delayed maxillofacial reconstructions are still very challenging. This study shows that custom ZrO_2_ implants can be used successfully in the late repair of extensive fractures in the orbital region.

## 5. Conclusions

Accurate shape corrections and bone reconstruction after craniofacial trauma can be achieved by modeling a titanium mesh on an individual anatomical model, subtractive methods (implants milled UHMW PE or PEEKs (Polyether ether ketone)) or additive methods (implants manufactured from metal powders). We propose the next accurate reconstructive option: skeleton reconstructions using osseointegrated, radio-opaque, personalized ceramic implants for craniofacial surgery. The treatment results are stable, and the therapy is safe.

## Figures and Tables

**Figure 2:**
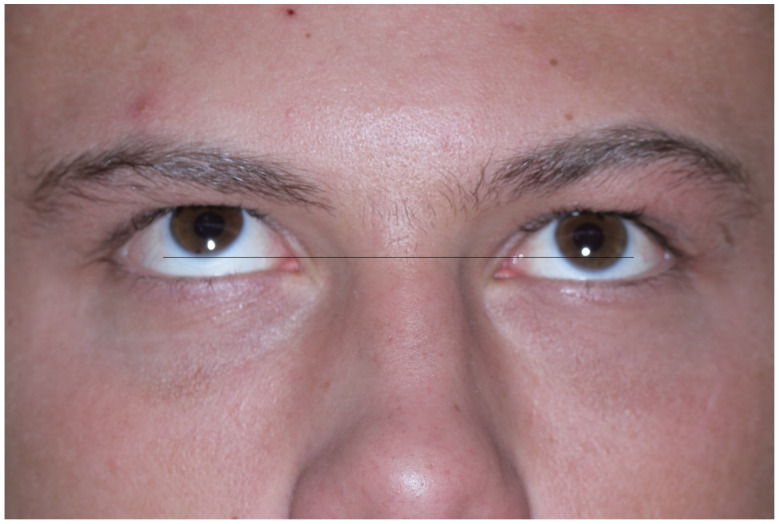
Patient 10 days after assault. Injury of the lower wall in the left orbit. Upgaze is limited in the left eye.

**Figure 3:**
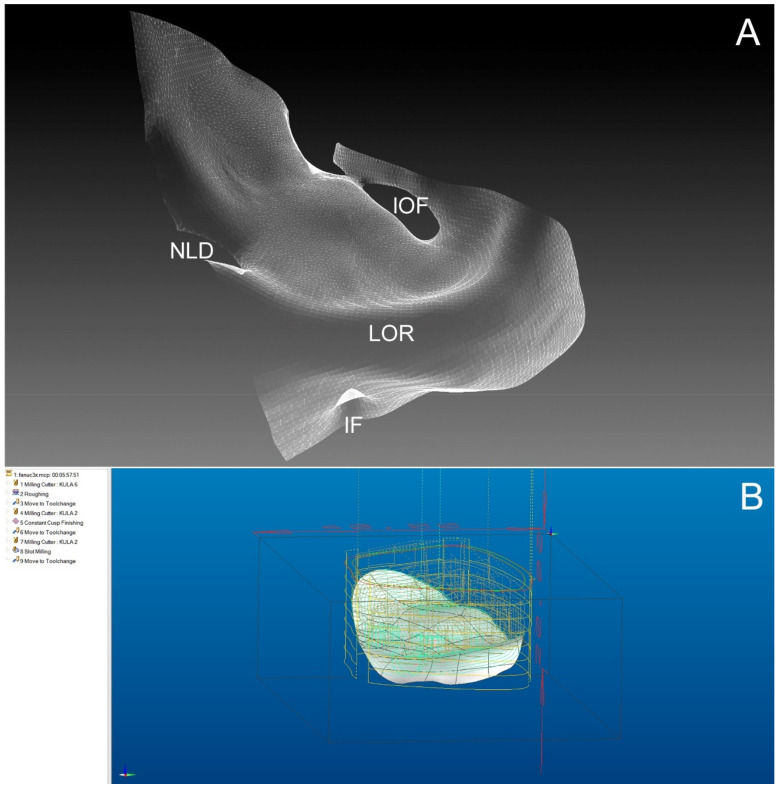
Segmented lower and medial orbital wall as shown by computed tomography. (**A**)—Creation of an intact orbit (IF—infraorbital foramen; IOF—inferior orbital fissure; LOR—lower orbital rim; NLD—naso-lacrimal duct). (**B**)—computer-assisted design (CAD). Design of custom implant-planning of the milling strategy for the zirconium oxide block. A 20% higher volume of implant is introduced.

**Figure 6:**
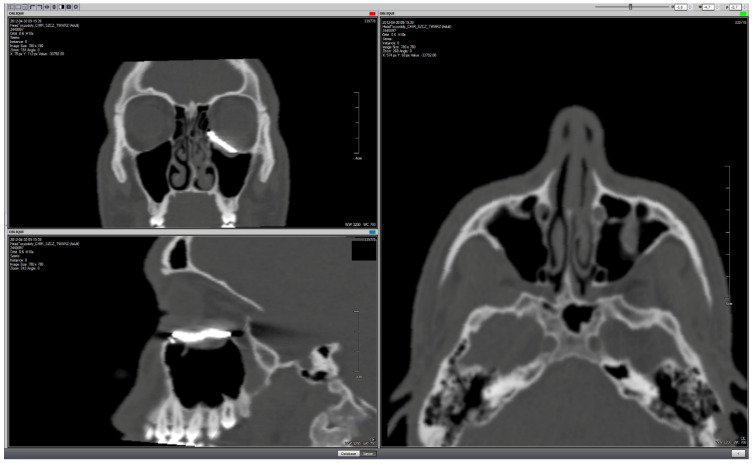
Postoperational computed tomography. Patient-specific implant reconstruction of the left orbital floor. Note: the artifacts around the implant (the real implant thickness varied from 0.46 to 0.71 mm, with a pseudo-thickness up to approx. 4 mm) caused by hardening of the X-ray beam due to the high molecular weight of the material.

**Figure 7:**
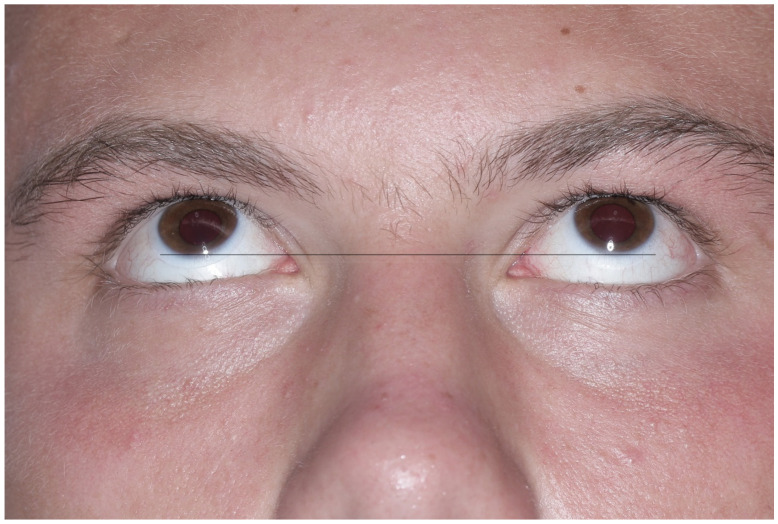
The patient utilized a patient-specific zirconium dioxide implant six months after surgery. Upgaze motility in the left eye globe was restored.

**Figure 8:**
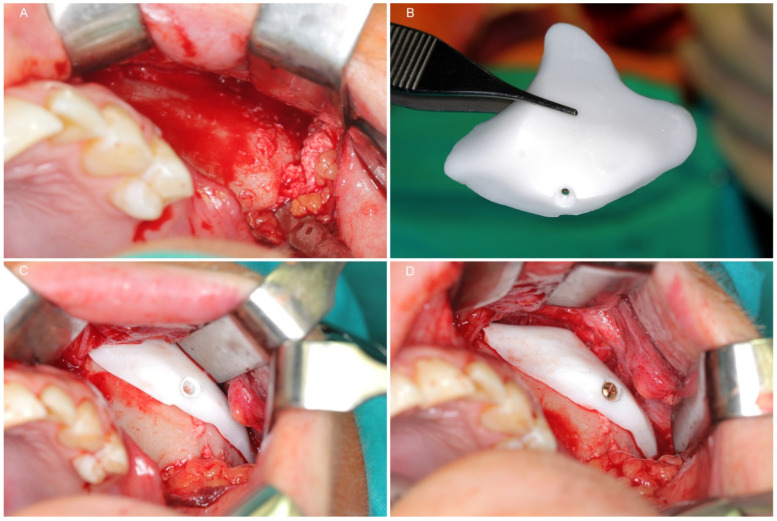
Application of the malar patient-specific implant. (**A**), Exposed surface of the left body of the zygomatic bone; this area is depressed because of an untreated zygomatic complex fracture. (**B**), Sterile zirconium dioxide implant ready to use. (**C**)—The implant covers the bone and reconstructs the symmetric convexity of the zygomatic body. (**D**), The implant is stabilized by one titanium microscrew.

**Figure 9:**
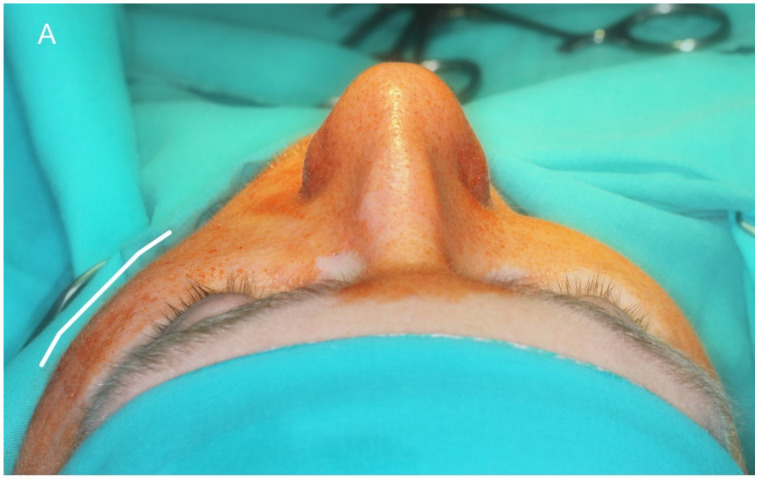

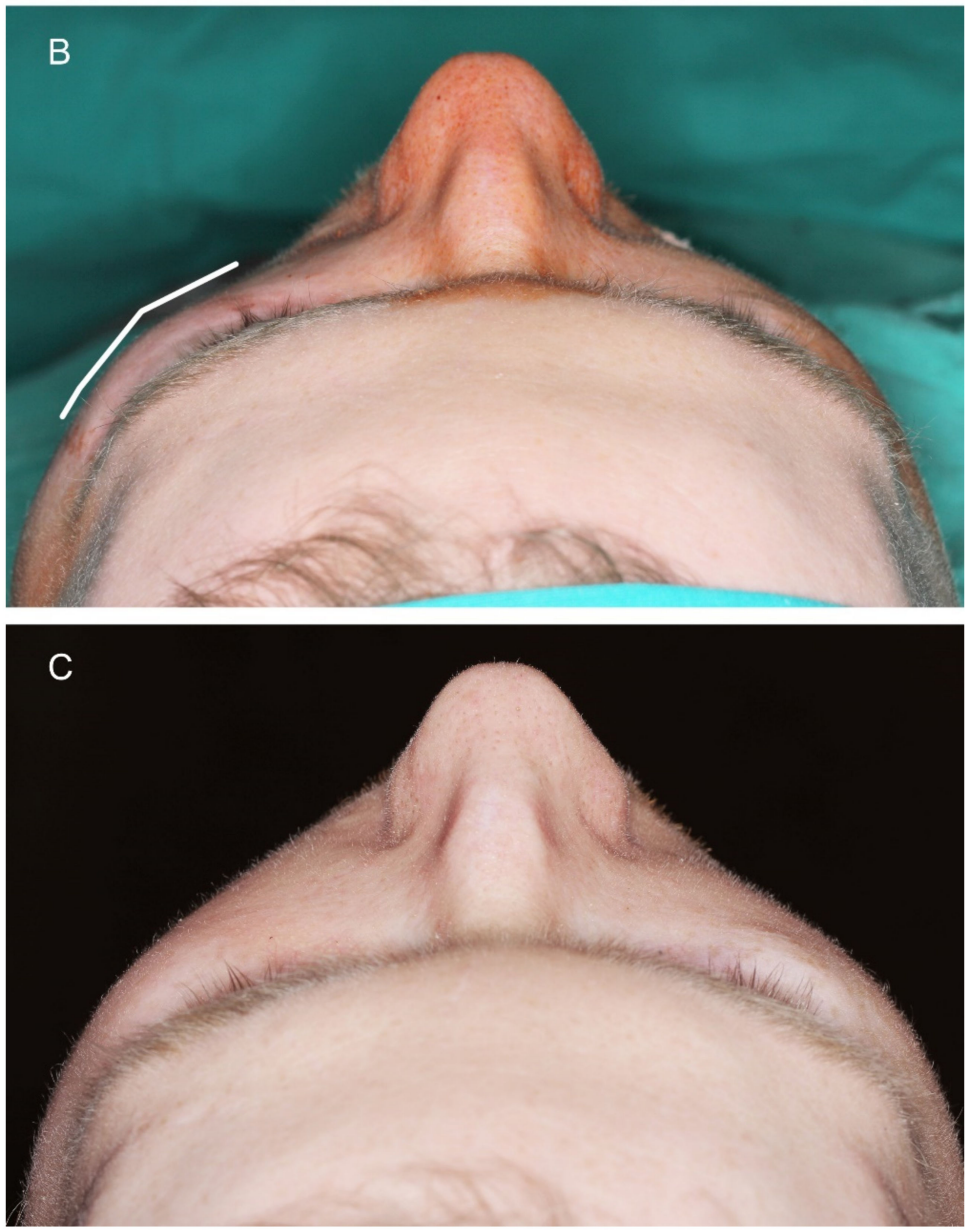
Results of the application of the patient-specific zirconium dioxide zygomatic implant. (**A**), In the left side zygomatic region, depression/flattening due to an untreated zygomatic complex fracture. (**B**), Immediate postoperative results: reconstructed zygomatic eminence on the left side. (**C**), One-year result showing stability.

**Table 1:** Cytotoxicity. Cell survival results in the lactate dehydrogenase test.

Material	Cell Viability ± SD [%]	Note
Control (−)	100 ± 5.6	NS
Titanium alloy	105.3 ± 7.2	NS
UHMW PE	102.9 ± 8.5	NS
ZrO_2_+ air-abrasion	101.2 ± 6.7	NS
ZrO_2_	103.6 ± 5.9	NS
Control (+)	12.5 ± 1.1	*p* < 0.05 vs. other materials

NS—not significant to other materials and Control (−).

**Table 2:** Genotoxicity. Number of micronuclei per 100 binuclear cells.

Material	Number of Micronuclei ± SD	Note
Control (−)	8.09 ± 0.91	
Titanium alloy	9.36 ± 3.10	NS
UHMW PE	8.56 ± 1.52	NS
ZrO_2_+ air-abrasion	12.51 ± 2.12	*p* < 0.01
ZrO_2_	11.18 ± 1.92	NS
Control (+)	22.53 ± 2.86	*p* < 0.001

NS—not significant to Control (−).

**Table 3:** Patient treated by zirconium dioxide custom-made implants.

Case	Sex	Age	ODI	Area of Reconstruction		Approach	Fixing Screws	Follow-Up [Months]	Soft Tissue Shift [mm]
1	M	19	1	Orbital wall defect	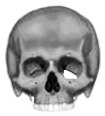	Tc	2	99	2.01
2	F	22	3	Orbital wall defect	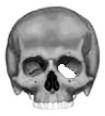	Tc	2	98	2.58
3	M	23	1	Orbital wall defect	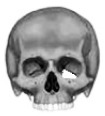	Tc	2	96	1.98
4	M	32	1	Orbital wall defect	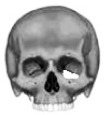	Tc	2	94	2.13
5	F	20	3	Orbital wall defect	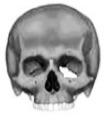	Tc	2	90	2.96
6	M	21	1	Orbital wall defect	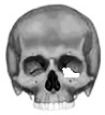	Tc	2	89	1.27
7	M	24	5	Orbital wall defect	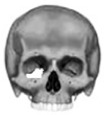	Tc	1	73	4.00
8	M	27	2	Zygomatic deformation	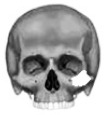	Tm	1	91	1.78
9	M	31	2	Zygomatic deformation	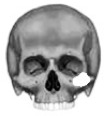	Tm	1	89	6.63
10	F	29	2	Zygomatic deformation	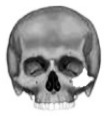	Tm	1	88	5.42
11	M	46	2	Zygomatic deformation	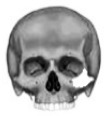	Tm	1	86	5.21
12	M	37	2	Zygomatic deformation	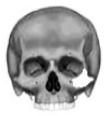	Tm	1	82	5.32
13	M	28	7	Orbital rim and forehead	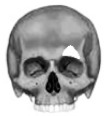	Se	2	82	3.89
14	M	24	7	Orbital rim and forehead	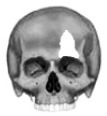	Sc	4	81	7.84

Abbreviations: F—female, M—male, ODI—orbital destruction intensity [15], Tc—transconjuctival, Tm—transmucosal, Se—skin eyebrow, Sc—skin coronal.

## Data Availability

Data sharing is not applicable to this article.
